# Compassionate Understanding in Dementia Care

**DOI:** 10.1007/s10728-026-00563-4

**Published:** 2026-04-07

**Authors:** Steve Matthews

**Affiliations:** Plunkett Centre for Ethics, Australian Catholic University, Melbourne, Australia

**Keywords:** Person-centred care, Personhood, Dementia, Compassion

## Abstract

Practitioners of person-centred care (PCC) for people living with dementia aspire to preserve and honour personhood through respect, relational care, and the promotion of agency and interests; yet its practical realisation often falters. This paper isolates an important moral-psychological element required for PCC’s fulfilment: the professional stance of *compassionate understanding*. Drawing upon philosophical analysis and empirical literature, the paper conceives this stance as a capacity combining enlightened compassion with four aspects of ethically informed care: forbearance, moral perception, skilled virtue, and empowerment. Compassionate understanding reconciles emotional attunement with the disciplined detachment required for professional endurance, thereby mitigating burnout while sustaining moral sensitivity and integrity. Grounded in an examination of dementia care practices, the paper argues that compassionate understanding constitutes an ethically desirable framework through which person-centred norms are realised.

## Introduction

Person-centred care (PCC) remains the central guiding ethical framework for dementia support, founded on recognition, respect, and the sustaining of personhood through care relationships. Yet its promise is frequently unfulfilled. Despite its influence, PCC often fails at the point of practice – not because its ideals are mistaken, but because the conditions for their realisation are absent. PCC is normatively well-motivated, but operationally fragile. This paper puts forward a suggestion for repairing this fragility: to support the moral psychology of practitioners so that the norms of PCC may be best achieved. For, merely knowing the principles of PCC is not enough; one must embody a professional stance capable of translating them into action, sometimes amid distress, confusion, and constraint. This paper argues for such a stance which I dub *compassionate understanding*.^1^

Compassionate understanding combines a disciplined form of enlightened compassion with four intersecting components – forbearance, moral perception, skilled virtue, and empowerment. When practiced, it restores a balance between detachment and emotional attunement, allowing carers to sustain both competence and care. As such it provides the correct bridge between the principles underlying PCC with their operation by care staff at the coalface.

The paper will proceed as follows. I first briefly recount the nature of dementia, particularly its stagewise progression in the context of dementia care (Sect. 2), and I then describe PCC and the difficulty in its operationalisation (Sect. 3). Section 4 describes the dilemma professional people face between becoming too detached or too emotionally affected, where it is argued that empathy is the wrong response to a resolution. This sets the stage for the introduction of compassionate understanding as the correct professional stance (Sect. 5). Finally, Sect. 6 clarifies the internal structure of this stance to respond to some possible objections.

## Dementia, and Its Stages

It will be useful, first, to briefly describe the relevant defining features of dementia, while noting two important propositions: first, dementia is progressive, developing through stages (typically called mild, moderate, and severe), and second, that the stance a carer takes (thus) depends critically on which stage a person has reached. At the mild stage, a person diagnosed with dementia typically experiences subtle and fluctuating symptoms, following earlier prodromal signs. As such, treating a person with less than full agency is potentially damaging, and stigmatising. In the early stages of dementia there is no good reason not to adopt a participant attitude, in contrast to an objectifying doctor-patient attitude.^2^

“Dementia” is an umbrella term that refers to a cluster of typically neurocognitive symptoms leading to progressive decline; there are a number of underlying pathological causes for dementia, chief among them being Alzheimer’s Disease, with some estimates suggesting it accounts for up to 80% of cases.^3^ Other common pathologies include the vascular dementias (5–10% of cases), frontotemporal dementias (around 10% of cases), and Lewy body dementia (around 5% of cases).^4^

Alzheimer’s disease (AD) is characterised by progressive impairment across six cognitive domains: attention, executive function, learning and memory, language, perceptual-motor abilities, and social cognition.^5^ Ultimately each of these domains may be affected, with AD following a recognisable trajectory starting with deficits in learning and memory accompanied by early executive dysfunction. AD, that is, has a typical “narrative” signature in its presentation over time. Cognitive losses cascade into a range of functional deficiencies: e.g., repeating the same story or information during one conversational encounter, forgetting names, losing track of lists, or tasks that require the retention of items that articulate with some daily operation, say following a recipe, completing a simple application, or making purchases. Executive function deteriorates alongside memory, undermining the capacity for sustained attention, cognitive control, and reflective, effortful reasoning required to manage complex or novel situations.

It is common to see the progress of dementia as delineated by mild, moderate, and severe stages, and the selfhood-relevant markers of these stages are important in the current context. The functional losses are specific to the different pathologies, so, given its predominance, we again should focus on AD. (In, e.g., behavioural variant Frontotemporal dementia, radical changes in personality are typical first signs; in Dementia with Lewy Bodies, visual hallucinations, sleep disturbances, and motor symptoms are early signs.)

At the mild stage in AD, individuals retain independence but tasks are made more difficult due to memory gaps. People may become withdrawn or irritable, experience a diminished sense of temporal coherence, a vagueness in self-understanding, with capacities for practical reason strained, but basically intact. During the moderate stage (usually the longest), people experience a breakdown in autobiographical continuity, marked by retrograde amnesia, confabulation, and some delusional states. There is loss of coherence between past, present, and future orientating beliefs and attitudes, with an increasing focus on the present. Individuals at this stage typically require help with daily activities – dressing, or cooking, say – and find social interaction, and conversation demanding, repeating themselves and losing track of things. During the severe stage, profound losses occur in thinking and communication; this decline is characterised by highly limited verbal capacity, loss of recognition of people and things, and near total dependence on others for self-care (bathing, eating, toileting). Eventually, there can be a loss in basic motor skills. These different stages are not rigid; nevertheless, they provide an ethically salient set of distinctions for thinking about how person-centred care must be tailored in ways that respect residual forms of selfhood.^6^

The fact that personhood and agency are sustained in relatively robust fashion through to the moderate stage of dementia importantly underwrites the possibility of person-centred care.

## Person-Centred Care, and the Failure to Operationalise Its Core Norms

Person-centred Care (PCC) began with the work of Tom Kitwood, drawing partly on Carl Rogers’ client-centred therapy, leading to what he called an enriched model of dementia [5, 6]. Kitwood ([6]: 8) emphasised that dementia is not merely neurological, but also psychological and social, and famously he defined *personhood* as a moral status “bestowed in the context of relationship and social being.” Good care, on his view, requires recognition, respect, and trust, and the avoidance of “malignant social psychologies” where residents are disempowered, infantilised, intimidated, or stigmatised.^7^ Another author, Dawn Brooker [7, 8], later distilled these insights in her influential VIPS framework – Value, Individuality, Perspective, and Social environment – emphasising that personhood is maintained only in relationships and through a supportive social world.

Building on Kitwood, I argue that care practices often misconstrue PCC by focusing too narrowly on individual personhood, autonomy, and consumer choice. Instead, care should aim to sustain *relational selfhood* – understood as an ongoing, grounded, co-authored sense of identity – rather than personhood in the abstract. Such a Relational Care Framework (RCF) emphasises how carers can promote continuity of selfhood through the different stages of dementia. A clear and instructive example of how this may occur involves carers enabling people living with dementia to continue to fulfil certain role identities that they may enact when a past self-image is preserved in the so-named “petrified self” cases.^8^ The RCF recovers Kitwood’s original vision and offers practical ethical guidance: carers adopt participant or quasi-participant stances when it is possible to do so – rather than managerial authority positions – that respect the person as a continuing self, while upholding dignity even when cognitive capacities wane significantly.^9^

Relational care, properly internalised, contributes to person-centred care, but for carers to fulfil their role, mere knowledge of the framework is not sufficient for effectiveness. Even those who are aware of the need for instituting PCC, including being in possession of an understanding of framework principles, can often fail to achieve its aim. These problems in operationalising PCC are revealed when we consider cases where staff and home carers lack the right training and experience.^10^

In order to motivate my account, then, it is necessary to see that PCC is insufficient to address the difficulties in dementia care unless it is accompanied with a moral psychology for carers, one which implements it in the full range of client-facing contexts. There are many obstacles to its implementation. I mention some representative ones here – agitation/aggression, restlessness/wandering, and delusional beliefs – and I propose that the stance of compassionate understanding is the best approach to addressing them.^11^

*Agitation*: In empirical work on agitation in long-term care, barriers to implementing the nonpharmacological strategies of PCC repeatedly surface. One recent multi-stakeholder synthesis [18] noted that although staff often recognise the value of tailoring care to the individual, the constraints and pressures of being a carer often led to defaulting to medications or containment as “easier” responses. In effect, practitioners find themselves torn between aspiring to achieve the ends of PCC, and the need to be pragmatic when situations become difficult to manage.

*Wandering*: The tension is also seen in encounters with so-called wandering or elopement.^12^ Ethical analyses in the dementia care literature characterise these as paradigm stressors for staff concerned to adhere to PCC protocols. Unfortunately, in attempting to reduce the risk – motivated out of the duty to protect – care staff will resort to restrictive measures, such as locking doors, setting exit alarms, or applying a variety of physical constraints. Each of these practices goes against good relational care, which would otherwise promote and encourage agency, and honour the dignity of risk, and for those striving to deliver PCC this can lead to moral injury and distress.^13^

*Delusions*: Delusional beliefs raise another set of stresses, especially in cases where, e.g., residents express desires that respond to these delusions, possibly engaging in risky behaviours, and agitation (This last point shows nicely that the different aspects of BPSD are not discrete; indeed, they routinely intersect). Consider the case of a woman who, in a common room filled with residents, insists that she is about to give birth. She begs the staff to get her to a hospital. Staff are faced with an immediate emergency – since the woman is now at physical risk – and a longer-term question about how to respond. Do they triage for pain and safety, avoiding a blunt contradiction of the woman’s belief in order to deescalate the situation? They clearly face a practical bind and accompanying stress, since either course of action potentially leads to harmful effects.^14^

These examples are not isolated, representing a widespread problem, which leads carers to report moral distress: they know what a person-centred response would be, but system constraints – time, culture, even liability fears – can seem to force them into responses (chemical restriction, physical restraint, curtailment of autonomy, unnecessarily frequent lying) that they regret.^15^ The emotional and ethical “residue” from this accumulates, leading potentially to career burnout, and staff shortages which further exacerbate the problem. It thereby indicates that PCC must be more than a set of techniques, and blind attempted application of guiding principles; it requires the kind of professional stance that builds in a moral psychology which properly equips staff to execute their roles over the long term.

## A Dilemma for Professionals is not Solved by Empathy

In the difficult cases we have just considered, detachment is called for, and carers who are unprepared or untrained in this approach, find professional life daunting. This forms the backdrop to a dilemma between being too detached on the one hand, or being subject to empathic distress on the other. It will help to understand this dilemma, because my argument is that compassionate understanding is the correct stance to negotiate it.

Here is the problem. Professionals who routinely encounter distress, grief, or suffering are vulnerable to emotional contagion – especially the tendency for sympathy. The philosopher David Hume ([23], 3.3.1.7) compared this tendency to the way stringed instruments naturally resonate in the presence of others. This is an involuntary empathic susceptibility, and while it helps explain our natural dispositions to be concerned for others, it can also impair judgment, disable concentration, and lead to losses in competence. Over time, empathic concern – defined, roughly, as sharing the perspective of another by feeling what they feel – may culminate in occupational burnout. To prevent this, the standard strategy is professional detachment, that is, the cultivation of psychological distance between professional and client, a technique aimed at preserving effectiveness and moral composure. Yet, detachment carries with it the risk of over-detachment, for it can desensitise, dull moral perception, and render professionals estranged from human connection and normative purpose, those things that are integral to virtue in the helping professions.

Thus, summed up, we have the following:

*Too little detachment* leads to emotional overload, empathic fatigue, and loss of competence.

*Too much detachment* produces coldness, proceduralism, and moral blindness – a failure to respond authentically to the suffering of others.

The central challenge is therefore to aim at achieving an equilibrium between virtuous caring engagement and emotional restraint. Some authors, e.g., Oakley and Cocking ([24]: 150), have responded to the problem by stressing the need for empathy, or empathic concern. But if what is meant by “empathy” is the Humean idea of feeling what another is feeling, this strategy is doomed to fail.^16^ That is because empathy of this kind leads to vicarious emotion-sharing which can be emotionally overwhelming over time. The response of compassionate understanding proffered here preserves moral sensitivity and motivation while insuring against the emotional burnout following moral distress or moral injury. This response reframes detachment, not as indifference, but as a disciplined moral capacity – a way of staying connected to professional values while safeguarding endurance and competence, and moreover, giving effect to its key virtue, enlightened compassion. Compassionate understanding enables carers to regulate, or calibrate, correct levels of detachment, in order to remain effective professionally, while emotionally protected.^17^

## Compassionate Understanding

Compassionate understanding for carers is constituted by a complex attitude combining the virtue of enlightened compassion (at its core) with a set of interacting abilities for moral understanding, viz., forbearance, moral perception, skilled virtue, and moral satisfaction. Once acquired, this attitude, and abilities, form into a professional *stance*, one that reconciles emotional attunement with the disciplined detachment needed for sustained, morally grounded, care. I present this as an alternative to empathy-based approaches that risk over-identification with client suffering because, as indicated above, they can lead to losses in professional competence, or worse the exhaustion seen in occupational burnout syndromes.^18^

Compassionate understanding is a stance with broad remit – it may be adopted in a large range of client-facing professions, especially those in which trauma, anguish, and distress are present. Given this broad application, I should emphasise the precise relation between it and PCC: it should be understood as an *independent normative precondition* for it, not an instantiation of its moral psychology as such. This is important since it suggests the stance is flexible across professions, which it is, and it suggests that PCC without the training to apply it, will not be effective. It also suggests the need for ongoing audits to ensure its longevity.

Thus, compassionate understanding is put forward as a complement to a professional ethic within client-facing caring occupations, and this places limits on its role. For, I construe it here as the moral psychology needed as a bridge between principle and practice. It is not, and could not be, sufficient to equip professional agents with an ethically complete set of tools for all situations. This limitation gives rise to at least two things: first, compassionate understanding is not sufficient to address all conflicts and dilemma situations that arise in professional settings, especially those that are novel; and second, given this limitation, organisations could not, and should not, rely on putting in place resources to institute compassion training, as if doing so will suffice for full accountability in relation to an organisation’s stated ethical mission. Typically, what must be in place in addition, therefore, is that institutions and the professionals within them, adhere to a more general code of organisational protocols, and this in turn may be justified by a more general theory of ethics.^19^

The stance itself consists of two interrelated elements: enlightened compassion and four components of what I am broadly describing as moral *understanding*.

*Enlightened compassion* refines the ordinary notion of compassion through reflective insight. Drawing partly on, and revising Martha Nussbaum’s Aristotelian [27] analysis, I argue that genuine compassion involves three elements: recognition of the seriousness of another’s suffering and a desire to relieve it; a compassionate concern that is unqualified by judgments of fault; and third, an impartial orientation that sets aside identity or in-group bias, and foregrounds an awareness of shared vulnerability. Enlightened compassion is thus an *unbiased*,* reason-guided desire to relieve serious suffering*, one that does not depend on feeling what the sufferer feels, but on grasping what their situation morally requires. For professionals, this form of compassion avoids empathic distress while sustaining moral motivation.

The moral *understanding* component of the stance comprises four intersecting/overlapping aspects:

1. *Forbearance* – carers displaying this quality show patience combined with a non-judgmental attitude when confronted with agitation, hostility, or despair, thereby allowing a space for calm decision-making. Its possession enables enlightened compassion, especially under conditions of emotional strain. In dementia care, remaining composed when a resident is agitated (say) is essential in the delivery of PCC when, by contrast, resorting to reactive judgement would be counterproductive. The carer who exercises forbearance does not merely suppress feeling but regulates it via the recognition that an aggressive outburst from a resident may simply reflect fear, disorientation, or cognitive loss, and does not indicate a personal hostility. Such patience allows the carer to maintain moral clarity and distinguish between surface behaviour and the person beneath. In this respect, forbearance functions as both a moral as well as an epistemic virtue, as it preserves the capacity to understand a resident, given the clinical history and context.^20^
2. *Moral perception* – this refers to a capacity for morally informed situational awareness, to perceive accurately what is at stake, integrating affective sensitivity with moral judgment.^21^ It is the capacity to identify what is morally salient in a given situation prior to, or independently of, explicit deliberation. Within dementia care, this allows practitioners to automatically detect suffering, or vulnerability, and to see what needs to be done in response. Such perception is neither purely cognitive nor purely affective, but an integrated moral attunement shaped by experience, training, and reflection. It involves perceiving not only the clinical facts of decline or dependency but also the moral demand implicit in another’s distress. In the context of compassionate understanding, moral perception thus provides the epistemic grounding for appropriate emotional regulation and moral responsiveness. It transforms the carer’s encounter with (say) distress from a merely procedural engagement into an ethically informed one. Moral perception, once refined through training and experience, forms the basis for the development of skilled virtue. The practitioner who repeatedly perceives and responds well to morally charged situations gradually transforms perceptual insight into embodied know-how, and motivation.
3. *Skilled virtue* – Skilled virtue in this context is the capacity to act compassionately through the seamless integration of moral perception and practical wisdom.^22^ Like other complex skills, it develops through a layering of habits and reflective practice until formerly effortful acts of discernment and regulation become spontaneous. In this sense, the skilled practitioner does not merely *apply* moral principles but *perceives* the moral contours of a situation directly, intuitively recognising what is called for and adjusting levels of detachment accordingly. Deliberative skill remains essential for novel or ambiguous cases, but as experience deepens, intuitive responsiveness predominates. Thus, compassionate understanding – where it imports skilled virtue – involves the refinement of moral vision through experience, training, and reflection, allowing the professional to act both efficiently and humanely. It shows how compassion, when disciplined through skill acquisition, becomes a reliable, embodied capacity rather than a transient emotion. Through the consolidation of skilled virtue, compassionate understanding becomes not only more reliable but also more self-sustaining. The capacity to respond with discernment and composure fosters a sense of agency and professional efficacy, paving the way for the rewards of practice – those forms of empowerment and satisfaction that arise when moral skill and humane purpose are harmoniously aligned.
4. *Empowerment* – this refers to the motivational reward accompanying skilled compassion, where helping and healing are sources of delight. This aspect echoes Aristotle’s idea of acting for the sake of *kalon*, or moral excellence, perhaps colloquially captured in the slogan ‘virtue is its own reward’. Once in place it can help to reinforce resilience and the ongoing commitment to client-facing work. The achievement of a truly virtuous disposition is accompanied by the absence of conflict between desire and (nobly) motivated action, wherein the performance of the latter is the very thing that brings pleasure. Aristotle discusses this explicitly in Book 1, Chap. 8 of the Nicomachean ethics [40]. The possession of *kalon* is empowering for professional carers because it embodies an implicit understanding that acting for the sake of a vulnerable person is never a hindrance, but on the contrary a deep professional and personal satisfaction, and it is so *because* it is morally good.

Compassionate understanding reliably equips carers to operationalise person-centred care, and this claim is tested when we consider examples of BPSD listed above. For example, faced with (seemingly) aggressive behaviour a professional person must, in the first instance, show forbearance in order to make competent judgments concerning its causes. A person with a delusional belief who is distressed may be in danger. The right stance here must combine situational awareness – what is the cause of the false belief, and what are the immediate risks? – with the desire to relieve the (potential) suffering. And the motivation to do so, and the action following in its wake, ideally will bring professional satisfaction. This stance, then, tracks processes of that are typical in the professional life of a carer concerned to fulfil principles of PCC.

## Clarifying the Connections Between the Four Components

Several questions and difficulties arise in relation to how the four components of the understanding aspect of the stance articulate, and how they may apply in different scenarios. In this section I address these questions.^23^

Before doing so, I should preface my remarks by emphasising that the four components interact and overlap both theoretically, as well as in their practical application; indeed, this is a defining feature of the stance. For example, a virtuously skilled carer must apply certain principles, e.g., acting justly by allocating fair time between clients or residents, while also seeing what is morally at stake in a situation – in terms of urgency and severity – precisely in order to be able to correctly apply the principle of justice that is in play. Thus, skilled virtue, in this case strongly overlaps with moral perception. In fact, there is an unambiguous intersection between the two.

That said, nevertheless, this general prefacing remark leaves several specific worries to be examined.

First, I have claimed that moral perception appropriately becomes automatic, but this may lead to worries about unreflective or uncritical action, and this could be exacerbated in dementia care where we find power asymmetries between staff and some residents. For example, a carer may make a judgement about a resident’s needs based on a perception which is framed partly in terms of a sense that their authority over the resident entitles them to determine the nature of those needs. What this shows is that moral perception ought to be sensitive *to the right framing*, even though, as a perception it carries with it the important quality of *rapidly* seeing what is at stake morally in the relevant situations. A degree of automaticity is a precondition for rapidly seeing what is required. Indeed, the absence of automaticity would be significantly ethically problematic in dementia care where timely responsiveness is needed for responsible care. For if every morally salient feature of a situation required deliberation, rather than some procedural fluidity the result would be delays, escalating distress, and increased risk.

But how can perception remain sensitive to the right moral framing of situations, as I have claimed above? Key to this is recognising that the faculty of moral perception does not form what cognitive scientists refer to as an “encapsulated module”; rather it is subject to “cognitive penetrability”. What does this mean? (Gerrans and Kennett [36]: 3–4) write:

The term cognitive impenetrability was introduced by Zenon Pylyshsyn to clarify a debate among cognitive scientists about the extent to which a person’s knowledge, goals and expectations could influence her perceptual experience and consequent judgements. Pylyshsyn pointed out that if perception could be influenced in this way the processes involved in representing goals, beliefs and expectations could not be independent of those involved in perception. As he put it, perceptual processes would be cognitively penetrable.

Given cognitive penetrability of moral perceptions, it is therefore possible that automatic responses are subject to influence in real time – they are dynamically responsive, and continuously updated. Automaticity in this context, should not be confused, then, with rigidity. Consider two possible cases, out of an indefinitely large number of situations carers face daily:

Example #1: Reframing seeming non-compliance. A resident (Rosie) refuses to take a shower. The carer (Carl) automatically attributes this to wilful non-compliance. But Rosie also seems fearful, and the carer then recalls that unfamiliar environments trigger distress. So, Carl’s perception shifts in the moment from seeing Rosie as resisting to seeing her as anxious. Carl responds accordingly based on a different principle of care.

Example #2: Reframing agitation. Carl hears a resident (Richard) shouting and automatically attributes this to agitation requiring some corrective measure. But upon seeing Richard frustrated and confused by a word-finding difficulty, Carl shifts to seeing Richard as someone in need, not as a potential threat. Again, Carl responds accordingly upon a different principle of care.

Second, a significant objection arises in connection with the way the components of moral perception and skilled virtue are related. These components seem to overlap, so that there appears to be theoretical redundancy. Yet if they are, in a certain sense distinct, what exactly *is* their theoretical relation, and how might their instantiation in practice be affected by these questions?

I have gestured above at an answer to this question, and here I want to tease out some further important aspects which will inform practice. As background, a key distinction is between the different *functions* of the components (what they do and what they are for), and the *phenomenology* in play (how they are experienced and felt) when moral perception and skilled virtue operate in practice. Consider phenomenology first. In very general terms, once the different components of compassionate understanding are incorporated the result is a seamless unity in the experience of delivering care in practice. (Compare this to the way sports people unify the components of practice into a seamless unity. It is almost a cliché in the world of sport that players must forget about the components of training when playing in real matches.) Thus, at the level of practice and phenomenology, the functions of the components are not made explicit.^24^ But at the theoretical level we must acknowledge and distinguish the various components. That is, functionally, upon reflection by others or oneself, the components are analysed as theoretically distinct and norm-governed in sui generis ways.

Given this background distinction between theoretical function and practical experience, we can then claim without contradiction that:

1/ During practice, a carer does not need to toggle between two different modes, “now I morally-perceive X”, and “now I virtuously act”. Rather, they simply operate professionally by utilising both capacities within a single process.

Whereas,

2/ During analysis, or training, debrief, etc., we must distinguish the different components; they play different epistemic, or motivational roles.

And now we can see, at the theoretical level, the non-overlap between these two components. In somewhat crude terms, moral perception has a direction-of-fit wherein *beliefs and judgements* are formed (world to word); skilled virtue has a direction-of-fit from belief to *action* (word to world). Moral perception, we might say, is for the purpose of generating accurate moral beliefs within a circumstance, and skilled virtue partly relies on these beliefs, or moral judgements, to be thereby morally motivated towards achieving a certain goal by acting within that circumstance. Astutely incorporating the components within a well-tuned moral psychology means that the overlap we see – especially at the phenomenological level – is not a defect within the framework, but an essential part of it.

A further reason to think these components differ is in terms of their stability. Moral perception is partly a function of external conditions, necessarily context-sensitive, and because of this dependence it is fallible. It is unstable in that sense just because individuals cannot fully predict and control the work environment. Skilled virtue, by contrast, is largely a function of a person’s internal makeup. It is hardy, and like a muscle it responds to training and repeat experiences. It is habitual, embodied, temporally extended, and robust under changing conditions. Indeed, cross-situational stability is a defining feature of virtue, and as Hursthouse and Pettigrove remark [37],

[Virtue] is a disposition, well entrenched in its possessor – something that, as we say, goes all the way down, unlike a [mere] habit such as being a tea-drinker – to notice, expect, value, feel, desire, choose, act, and react in certain characteristic ways.

The philosopher Peter Railton [38] has usefully compared virtue-based action to skills generally. Again, consider professional sports people: even on bad days when they are below their best, their form will be largely maintained, and they will still “react in certain characteristic ways”. Skills are robust and stable, *ceteris paribus*.

To sum up the overlap issue: essentially, moral perception and skilled virtue play distinct functional roles yet must be brought together seamlessly in practice. Moral perception is *epistemic*, involving the capacity to discern what is morally salient in context. Skilled virtue, by contrast, forms part of the chain of practical reason and *agency*.

Third, the component of empowerment – based on Aristotle’s notion of *kalon* – is put forward to explain why it is desirable for professionals to align their desires with moral motivations. Aristotle’s thought is that when we desire what we ought to do, and perform accordingly, we derive satisfaction. It is an ideal, to be sure, and one which almost all of us do not achieve all of the time, though some of us achieve it at least some of the time. As an ideal, I do not claim that it should form a necessary part of compassionate understanding. Rather it is permanently potentially *compatible with* a virtuous stance, and directs the individual carers who are subject to its norms to internalise them to a degree which achieves good results. Thus, empowerment remains as a potential internal good of the practice. A commitment to compassionate understanding does not carry with it the belief that carers will inevitably experience delight in their job – that would be absurdly optimistic – and so we should say that Aristotle’s idea remains as a conditional good. But we can also bolster this with the observation that *kalon* may emerge the more compassionate understanding is supported in the workplace.

To conclude this section: the four elements of compassionate understanding are analytically distinct, given their different functional and norm-governed aspects. In practice they do not operate sequentially or independently, but as mutually supporting aspects of a single professional stance. And to emphasise: the phenomenological unity described above should not be mistaken for normative opacity, or a sense that practitioners operate uncritically. The fact that moral perception and skilled virtue *can at times* tend to operate without conscious deliberation would reflect the *success* of training and habituation, not the bypassing of ethical reflection.

## Conclusion

At the core of the stance I have set out is enlightened compassion – the disposition to care for people in need – which is, on reflection, precisely what *should* be at the centre of any caring profession. The conditions of dementia care are often epistemically fraught, that is, the intentions, meanings, and values of people with dementia are often hidden, or unclear, and so forbearance and detachment are needed to maintain compassion, while determining what is needed quickly and accurately, and to respond with professional virtue. To understand what is really causing someone to act often means that a carer is in best position to address it – agitation may be caused by distress, refusal to do something caused by fear, and so on – and this helps carers to step back and properly calibrate their responses based on the professional principles that respect people as part of PCC. The process of compassionate understanding is thus a way of upholding the dignity of people with dementia while recognising the challenges they face under conditions of cognitive decline.

In summary, compassionate understanding is the stance needed at the interface between on-the-ground reality of care situations and the principled understanding that person-centred care defines. It is the “translation space” for these two aspects of care. It is where carers have power and discretion to apply the principles of PCC. In addition, compassionate understanding, and its training, underwrites professional endurance, and such endurance is critical to fulfilling professional careers and to enable longevity in staffing which is an important key to maintaining the relationships that form the backbone of PCC, and in particular any *relational* care framework. Moreover, in compassionate understanding, the carer’s motivation is to shape the interaction with a resident in ways that best achieve the moral aims of PCC, which after all, is the most fundamental aspect of dementia care.

## Data Availability

No datasets were generated or analysed during the current study.

## Footnotes

1 To forestall a possible objection: it has been put to me that training staff in compassion for dementia care is a luxury which is unaffordable and not really feasible. But compassionate understanding should not be interpreted as *presupposing* ideal levels of resourcing or organisational culture; nor is it a discretionary addition to practice, it is a *way* of delivering PCC. Certainly, adequate funding, staff competence, and collaborative interdisciplinary teams conduce to PCC, but the stance I advocate does not have to depend on this. Moreover, it can itself contribute to the development of supportive cultures, lead to more effective use of resources, improve staff retention, and reduce moral injury and distress. So, I think that to treat compassionate understanding as a kind of luxury misconstrues its role within care practice: it is best thought of as potentially part of an organisation’s ethical culture, and something through which dementia care systems can be made more humane, resilient and ultimately more sustainable.

2 The participant attitude – often called participant ‘stance’ in secondary literature – is due to Peter Strawson [1]; it is the idea that in relations with others our default is a proneness to respond to them with good or ill will. Strawson suggested that the ‘reactive attitudes’ within this stance – e.g., resentment, hurt feelings, love, or forgiveness – presuppose an ascription to others of human capacities for agency, and reason, and a default system of human relations structured by a set of interacting normative expectations. When we cease reacting to others this way – moving to hold what Strawson called ‘objective attitudes’ – these presuppositions are suspended, e.g., when interacting with small children, or with people with severe mental illnesses who have lost the relevant capacities that normally underwrite default interactive participation.

3 This is a Centres for Disease Control (CDC) figure (see Alzheimer’s Disease and Dementia | Alzheimer’s Disease and Dementia | CDC), and is very much at the upper end. Other estimates, e.g., the World Health Organisation, put it at between 60 and 70% (see Dementia).

4 These figures derive from popular websites like those above. They do tend to vary depending on diagnostic criteria, misidentification of disease subtypes, geographical and socioeconomic factors, age/gender, and underdiagnosis due to stigmas and other social factors. I think a reasonably stable indication for disease frequency comes from brain bank data, and it is worth consulting a highly cited paper by Barker et al. [2] who did a study based on the State of Florida brain bank of 382 subjects. This revealed a breakdown of Alzheimer’s Disease (77%), followed by Lewy Body Disease (26%), Vascular Dementia (18%), Hippocampal Sclerosis (13%), and Fronto Temporal Dementia (5%). The non-addition to 100%, is explained by a relatively high proportion of mixed pathology, especially where AD co-occurred with most other conditions. It is of strong interest that Alzheimer’s Disease is easily the most highly represented pathology using this study method, and which I hope justifies its use as fundamental to considerations of stagewise care.

5 See DSM-V-TR [3]. Alzheimer’s Disease *causes* mild or major neurocognitive disorder. The term ‘dementia’ is eschewed there.

6 Joan Tronto [4] advocates a care model structured by four interrelated phases – caring about, taking care of, care-giving and care-receiving. These align with attentiveness, responsibility, competence, and responsiveness. This framework does not map on to the stages of care in dementia in a linear fashion, but nevertheless offers remarkable illumination for how care might shift across these stages. Early stage dementia requires attentiveness; moderate stage dementia demands competence; and later stage dementia requires an acute responsiveness to how care is experienced by individuals with significantly attenuated capacity. I thank an anonymous referee for alerting me to this connection.

7 See Tom Kitwood [6], esp. Chapter 4, *passim*.

8 See Daniel [9]; Oliver Sacks [10], retrieved 1/20/2026. Sacks refers to the case of Mr Q, a former janitor who came to believe that in the care home he occupied he *was* the janitor – his role-capacities were preserved. His carers, in kindness, and respect for his agency, enabled this role to continue there.

9 For an account of the difficulties in negotiating interactions with clients when their cognitive impairments undermine this stance, see Kennett [11]. The difficulty is acute when an individual oscillates between being in a competent state at one moment in time, then quickly losing capacity a short time later (possibly over periods as short as minutes). Care under these conditions is extremely fraught, and it partly motivates the need for managing the right level of professional detachment, as discussed in a later section. It is also partly what motivates the present work.

10 I will refer to the importance of compassion training throughout the paper. It comes via accredited and non-accredited programs. Two examples of the former: First, *Compassion Cultivation Training* is offered through Stanford University in the department of Psychiatry and Behavioural Sciences. (See Compassion Cultivation Training for Physicians and Psychologists | Department of Psychiatry and Behavioral Sciences | Stanford Medicine). And in Australia, Monash University offers a structured online course in Compassion Training for HCWs and educators. (See Compassion_training_for_healthcare_workers_DIGITAL-Debbie-Ling.pdf). There are a range of non-accredited, but serious, programs as well, including *Mindful Self-Compassion*, created by Kristin Neff and Christopher Germer, and *Compassion Focused Therapy* and its related *Compassionate Mind Training*, developed by Paul Gilbert. There is a limited but promising evidence base for the success of compassion cultivation programs within the health sector. See, e.g., [12, 13, 14, 15].

11 The three obstacles just mentioned are usually categorised as part of the set of behavioural and psychological symptoms of dementia (BPSD); they are posited here to be *representative* of those symptoms. I do acknowledge a controversy that has arisen concerning whether BPSD emerges directly from a brain pathology in dementia, or as an artifact of the care environment, or some interacting combination of both of these. My own position is that when care environments fulfil to the largest degree the moral aspects of PCC, BPSD reduces dramatically. This is especially pronounced when staff-to-resident relationships are nurtured. See Lee et al. [16], and Soofi [17].

12 The causes of this behaviour are elusive, including biomedical, psychosocial, or simply a rational outcome of occupying a certain environment, as Agrawal et al. [39] (p. 54) note. Some authors Sabat ([19], Chap. 4 *passim*) call for a shift: to talk not of wandering, but about ‘people who like to walk,’ and instead of forbidding or preventing movement, to design spaces for the expression of mobility.

13 See, e.g., Warren [20].

14 The case is adapted from Matilda Carter [21].

15 See Wendy Dean et al. [22]. This text is the *locus classicus* in the healthcare literature for the argument linking distress to moral injury.

16 See Bloom ([25], chapter 1 passim), who targets this conception. Van zly ([26]: 75) refers to a conception in which empathy involves a ‘suffering with’, which would problematise its professional application even more.

17 A question arises around what the correct levels of detachment are, given certain contexts and cases. See [author article].

18 The Maslach Burnout Inventory is the gold standard measurement tool. See Maslach Burnout Inventory (MBI) - Assessments, Tests | Mind Garden - Mind Garden.

19 In the author’s experience this ‘general theory of ethics’ is rarely explicitly articulated, but what is implied (within organisations devoted to care like hospitals and aged care facilities) is usually elements of utilitarianism, neo-Kantianism, and virtue theory (with an emphasis on the virtues associated with care, including compassion.)

20 Forbearance is of course not needed absent situations where BPSD are in play. As discussed earlier, in mild dementia, interactions need not diverge from the participant stance.

21 The concept of moral perception has been discussed by philosophers. See Audi [28], Crow [29], Harman [30], and Ross [31].

22 There is a burgeoning philosophical literature devoted to the skill model of practical wisdom. See Annas [32], Stichter [33], Swartwood [34] and Tsai [35].

23 I thank two anonymous referees for raising these problems which have motivated the present response.

24 I have elsewhere [41] discussed this at length in relation to the ‘flow’ states made famous by the psychologist Mihaly Csikszentmihaly.
